# One patient, two lesions, two oncogenic drivers of gastric cancer

**DOI:** 10.1186/s13059-014-0444-9

**Published:** 2014-08-27

**Authors:** Clara Alsinet, Marco Ranzani, David J Adams

**Affiliations:** Experimental Cancer Genetics, Wellcome Trust Sanger Institute, Hinxton, CB10 1SA UK

## Abstract

Deep-sequencing of a primary tumor and metastasis from a single patient, and functional validation in culture, reveals that *TGFBR2* and *FGFR2* act as drivers of gastric cancer.

## Introduction

When talking about cancer genome studies, big does not necessarily mean beautiful, and, as illustrated by Nadauld and colleagues [1] in this edition of *Genome Biology*, the careful analysis of just one gastric cancer patient can provide profound insights into the drivers of tumorigenesis. Recent estimates of the worldwide gastric cancer burden show that it is the fifth most commonly diagnosed cancer, and the third-leading cause of cancer death [2]. Current treatment options for early disease include surgical resection together with neoadjuvant or adjuvant chemotherapy, but five-year survival is only <60% [3]. Patients with advanced disease are treated with chemotherapy or chemoradiotherapy and show a median survival of around 12 months, whereas patients with a high level of expression of *HER2* (also known as *ERBB2*, encoding receptor tyrosine-protein kinase erbB-2) receive adjuvant trastuzumab (monoclonal antibody against HER2) and can be expected to have a median survival of 16 months [3]. Overall, gastric cancer is a disease with a dismal prognosis. When analyzing cancer genomes, our inclination has always been to sequence large numbers of tumors so as to draw the landscape of mutations. The deluge of data that ensues makes it difficult to read the life-history of each cancer or to appreciate the stories they may tell.

Here, Nadauld and colleagues [1] report the identification of candidate oncogenic drivers by undertaking exome and whole-genome sequence analysis of tumors from a single patient, followed by robust experimental validation. The patient they studied presented with a diffuse gastric cancer and a family history of the disease. Germline sequence analysis of *CDH1* (encoding the adhesion protein cadherin-1), a known driver, revealed an essential splice-site mutation co-segregating within the pedigree. Subsequent sequencing of a primary tumor from this patient and of a metastatic lesion revealed several drivers - an amplification of the *FGFR2* locus (encoding fibroblast growth factor receptor 2) that was found exclusively in the primary, and a deletion in the gene *TGFBR2* (encoding TGF-beta receptor type-2) found exclusively in the metastasis. In this paper, it was not so much that the authors set out to tell a story of gastric cancer - rather, the complete story of this cancer and its evolution, and make it a good read.

## The power of *N* = 1

In the world of clinical trials, the value of *N* = 1 (also known as ‘N of 1’) studies has long been appreciated. In this scenario, brave souls who are not yet ‘done’, or who are unwilling to accept their fate, step forward to try new experimental therapies. Albert Alexander, for example, was the fourth patient to receive penicillin from Howard Florey, and the profound anti-bacterial response it elicited was key to its clinical development. Likewise, dramatic responses in single patients treated with cancer therapies such as inhibitors of the serine/threonine-protein kinase B-raf (BRAF) and cytotoxic T-lymphocyte protein 4 (CTLA-4) have been key [4]. Yet, as cancer genome scientists, as soon as we could drown ourselves in data, we did.

**Figure 1:**
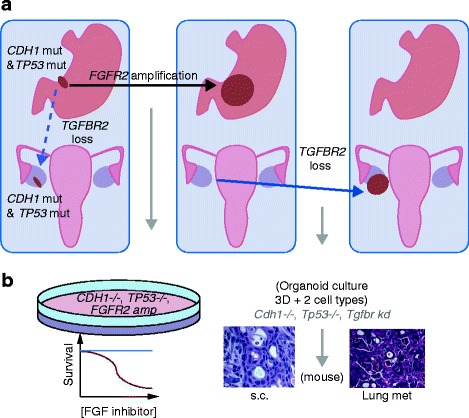
**Telling the story of a gastric cancer through sequencing and functional studies. (a)** The primary gastric cancer clone carried *CDH1* and *TP53* mutations. After it had metastasized to the ovary, the primary gastric cancer acquired an amplification of the *FGFR2* locus (black arrow). At this point, the primary tumor was removed by gastrectomy. Some three years later, the patient presented with abdominal masses. Sequencing of the metastatic tumor revealed loss of *TGFBR2*, but no amplification of *FGFR2*. Loss of *TGFBR2* might have occurred after the clone had spread to the ovary (non-broken blue arrow) or alternatively might have occurred as the metastatic clone transited from the primary to the ovary (broken blue arrow). **(b)** A schematic representation of the functional validation of candidate oncogenic events. A human gastric cancer cell line harboring mutations in *CDH1* and *TP53,* and an amplification of *FGFR2,* shows a significant reduction in survival in response to inhibition of the FGF pathway. Murine gastric organoids in which *Tgfbr2* is suppressed by knockdown (kd) in the context of *cdh1* and *Tp53* loss undergo oncogenic transformation *in vitro* when grown subcutaneously (s.c.) in immunodeficient mice*.* The resultant tumor shows histology consistent with diffuse gastric cancer and undergoes metastatic spread to the lungs. Abbreviations: CDH1, cadherin-1; FGF, fibroblast growth factor; FGFR, fibroblast growth factor receptor; TGFBR2, TGF-beta receptor type-2; 3D, three-dimensional; TP53, cellular tumor antigen p53.

So what do the mutations identified in the tumors from this gastric cancer patient actually tell us (Figure 1)? First, as a germline *CDH1* mutation was found, it tells us that an early chapter in the story was loss of the wild-type allele of this gene, probably coinciding with biallelic loss of the *TP53* gene (encoding cellular tumor antigen p53) or preceding it. Thus, mutation of *CDH1* and *TP53* are early events that probably drove tumor initiation and set the ball rolling. But why does the metastasis lack the *FGFR2* amplification? This is most likely explained by the early dissemination of tumor cells from the primary lesion, which was removed by gastrectomy at presentation, to the metastatic site in the ovary, with these cells lying dormant, only to be reanimated years later by loss of *TGFBR2*. An alternative scenario could be that loss of *TGFBR2* helped a cancer cell transit from the primary tumor to the metastatic site, where it lay in wait - either way, distal dissemination of the tumor must have been an early event occurring before removal of the primary.

This result challenges the dogma that metastasis occurs late during cancer progression and suggests strategies that kill occult cancer cells where they lie are important adjuvants to surgery. Importantly, the early dissemination of tumor cells poses some challenges in the clinical management of advanced disease. First, although genomic analysis revealed that inhibitors of *FGFR2* could be used to treat the primary lesion, they are unlikely to elicit a response in the metastasis. As it is the metastatic abdominal mass that represents the highest risk for the patient, treatment on the basis of changes found in the genome of the primary would represent wasted intervention time. This case had just one primary and one metastasis; some cases have dozens of metastatic lesions or synchronous primary tumors, compounding the challenge of personalizing cancer therapy. It appears that there can be no half-measures in profiling a patient’s disease. The complexity of cancer evolution does not make personalized therapy hopeless, just challenging, and it is only through the kind of molecular cartography described by Nadauld and colleagues [1] that we will be able to understand this disease.

The value of *N* = 1 cancer-genome studies is soon to be further tested as efforts to analyze the genomes of ‘exceptional responders’ - those patients whose response to therapy marks them as both lucky and different - gather pace. One particularly startling example of this approach was the sequencing of the genome of a single small-cell lung cancer patient, resulting in the identification of a *RAD50* mutation as a factor contributing to the patient’s profound curative response to combination chemotherapy (AZD7762 - an inhibitor of the serine/threonine-protein kinases Chk1 and Chk2 - and irinotecan, an inhibitor of topoisomerase 1) [5]. Most oncologists have stories of similar patients who, by all reckoning, should not have survived their disease; with sequencing, we will understand why, and this will help sculpt treatments for all patients.

## The right model of cancer, not just any model

Oncogenic transformation is a complex multistep process, and the precise number of alterations required is still an on-going debate. Nevertheless, it is widely accepted that, in most cancers, multiple hits contribute to the final tumorigenic phenotype. The identification of sets of alterations that cooperatively induce transformation is crucial in order to interrogate candidate oncogenic drivers in the appropriate genetic context. Here, Nadauld and colleagues perform all their validation experiments in the context of loss of *CDH1* and *TP53*. First, by using the gastric cancer cell-line KATOIII, which carries an amplification of *FGFR2* in addition to *CDH1* and *TP53* loss, they show response to inhibitors of FGF pathway tyrosine kinases and a therapeutic avenue that is potentially efficacious for the primary tumor. Notably, however, inhibitors of *FGFR2* have not been formally approved for gastric cancer. Second, an organoid-based model, discussed below, is used to test *TGFBR2* as a tumor suppressor and to assess its metastatic potential. Importantly, Nadauld *et al*. did not choose any model - instead, they carefully selected the right models, with functional validation of driver mutations being an integral part of the story, rather than an afterthought or footnote performed with ‘reviewer 3’ in mind.

## Organoid-based 'cancer engineering' as a validation strategy

Next-generation sequencing studies have flooded the literature with lists of candidate cancer genes, but these are just the ‘table of contents’ to the story of a cancer type [6]. The real story unfolds with functional studies that investigate how each individual cancer is wired. In addition to the cell-line studies described above, Nadauld and colleagues use a novel three-dimensional *in vitro* organoid model that consists of primary epithelial and mesenchymal gastric cells. As recently published by the same authors [7], this model allows for *in vitro* 'cancer engineering' through transfection of candidate or known oncogenic drivers into murine primary wild-type cells from the tissue of interest. Additionally, the presence of both epithelial and mesenchymal cells, and the three-dimensional nature of these organoids, accurately reproduces the complexity of the tissue of origin and its histopathology. As mentioned above, the *in vitro* oncogenic transforming potential of downregulation of *Tgfbr2* was tested on a background of loss of *Cdh1* and *Tp53* (*Cdh1*^*-/-*^*; Tp53*^*-/-*^), with histopathological characterization of the resulting organoids revealing a key role for *Tgfbr2* downregulation in a diffuse gastric cancer phenotype. Interestingly, disaggregated *Cdh1*^-/-^; *Tp53*^-/-^ organoids expressing small hairpin RNA (shRNA) targeting *Tgfbr2*, when injected subcutaneously into immunodeficient mice, showed increased tumor growth compared with *Cdh1*^-/-^; *Tp53*^-/-^ control cells.Furthermore, the distal spread of cells from the injection site into the lungs of the mice was observed, thus confirming the metastatic potential of the *Cdh1*^-/-^; *Tp53*^-/-^; *Tgfbr2* knockdown cells (Figure 1).

## The route ahead via *N* = 1

At heart, many of us are reductionists seeking to understand simple questions through the lens of genome sequencing. Just as the *N* = 1 clinical trial has profoundly informed us of new paths to therapy, the *N* = 1 cancer genome will increasingly be used to identify the key molecular factors that drive cancer evolution and mediate response to therapy. Recent studies, for example, have used *N* = 1 approaches to define new driver mutations and mutational signatures in melanoma and to hunt down the clone of origin for a lethal prostate cancer [8-10]. Nadauld and colleagues further illustrate the power of an *N* = 1 analysis - the advantage of combining these endeavors with functional studies and the value of every cancer patient’s story.

## Abbreviations

BRAF: Serine/threonine-protein kinase B-raf
CDH1: Cadherin-1
CTLA-4: Cytotoxic T-lymphocyte protein 4
FGF: Fibroblast growth factor
FGFR: Fibroblast growth factor receptor
HER2: Tyrosine-protein kinase erbB-2
TGFBR2: TGF-beta receptor type-2
TP53: Cellular tumor antigen p53
